# Birt–Hogg–Dubé syndrome presenting with macroscopic pulmonary cyst formation in a 15‐year‐old

**DOI:** 10.1002/rcr2.610

**Published:** 2020-06-24

**Authors:** Luke Ardolino, Elizabeth Silverstone, Vincent Varjavandi, Deborah Yates

**Affiliations:** ^1^ Department of Medical Oncology The Kinghorn Cancer Centre, St Vincent's Hospital Sydney NSW Australia; ^2^ Department of Radiology St Vincent's Hospital Sydney NSW Australia; ^3^ Department of Thoracic Surgery Sydney Children's Hospital Sydney NSW Australia; ^4^ Department of Thoracic Medicine St Vincent's Hospital Sydney NSW Australia

**Keywords:** Birt–Hogg–Dubé syndrome, cystic lung disease, familial cancer syndrome, paediatric lung disease, pneumothorax

## Abstract

Birt–Hogg–Dubé (BHD) syndrome is a rare, autosomal dominant disorder caused by a germline mutation in the folliculin gene (17p11.2). It is characterized by benign skin lesions, renal tumours, and pulmonary cysts, with pneumothoraces seen exceptionally rarely in patients younger than 40 years. We report the case of a 15‐year‐old boy who presented with sudden onset left‐sided chest pain and acute dyspnoea secondary to a large left‐sided pneumothorax. This failed to resolve despite chest drain insertion and he required video‐assisted thoracoscopic surgical pleurodesis, which revealed macroscopic pulmonary cyst formation. Following this, he made a good recovery and a further high‐resolution computerized tomography (CT) scan of his chest identified multiple, small, subpleural parenchymal lung cysts that were not initially visible on prior imaging. Further questioning revealed a strong family history of spontaneous pneumothoraces and additional genomic sequencing, and confirmed a diagnosis of BHD syndrome. We highlight the diagnostic, management, and surveillance challenges for this rare syndrome.

## Graphical abstract

Birt–Hogg–Dubé syndrome is a rare, autosomal dominant disorder characterized by benign skin lesions, renal tumours, and pulmonary cysts. Individuals with this syndrome have a sevenfold increase in their risk of renal cancers and over 80% will develop pulmonary cysts, of which ~30% will experience a spontaneous pneumothorax in their lifetime.

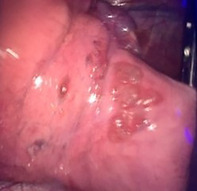

## Introduction

BHD is an autosomal dominant disorder caused by a germline mutation in the folliculin (FLCN) gene on chromosome 17 (17p11.2), which codes for the protein folliculin. The condition is characterized by pulmonary cysts, benign skin lesions, and renal tumours, although these manifestations are highly heterogeneous. The exact role of FLCN is unclear, but it probably functions as a tumour suppressor gene (TSG), preventing uncontrolled cell growth and proliferation via the mammalian target of rapamycin (mTOR) pathway [1]. As a result, individuals with BHD syndrome have an approximately sevenfold increase in their risk of renal cancers, amounting to a lifetime incidence of ~20% [2]. Additionally, pulmonary cysts occur in over 80% of individuals with BHD, of which ~30% will have a spontaneous pneumothorax in their lifetime [3].

## Case Report

A previously fit and well 15‐year‐old boy presented with sudden onset left‐sided chest pain and breathlessness while swimming. He had no significant medical comorbidities and reported no known drug allergies. He denied any history of tobacco or illicit drug use. He was born in Australia, had never travelled overseas, and currently was living at home with his parents and two siblings who were all in good health. He denied any recent infective or irritant exposures and was currently attending high school. On further questioning, there was a strong family history of spontaneous pneumothoraces, in both his mother and grandmother. On review of systems, he denied any fevers, cough, haemoptysis, weight loss, or other systemic upset in the preceding few months. On admission to hospital, the patient was afebrile, with a heart rate of 95 beats/min, blood pressure (BP) of 105/68 mmHg, respiratory rate of 28 breaths/min, and oxygen saturation (SaO_2_) of 97% on room air. He did not appear overweight, although body mass index (BMI) was not measured. On physical examination, salient findings included absent air entry, hyper‐resonance on percussion, and reduced chest expansion over the entire left side of his chest. His examination was otherwise normal, with no heart murmurs, clubbing, cyanosis, organomegaly, skin lesions, or musculoskeletal abnormalities identified.

**Figure 1:**
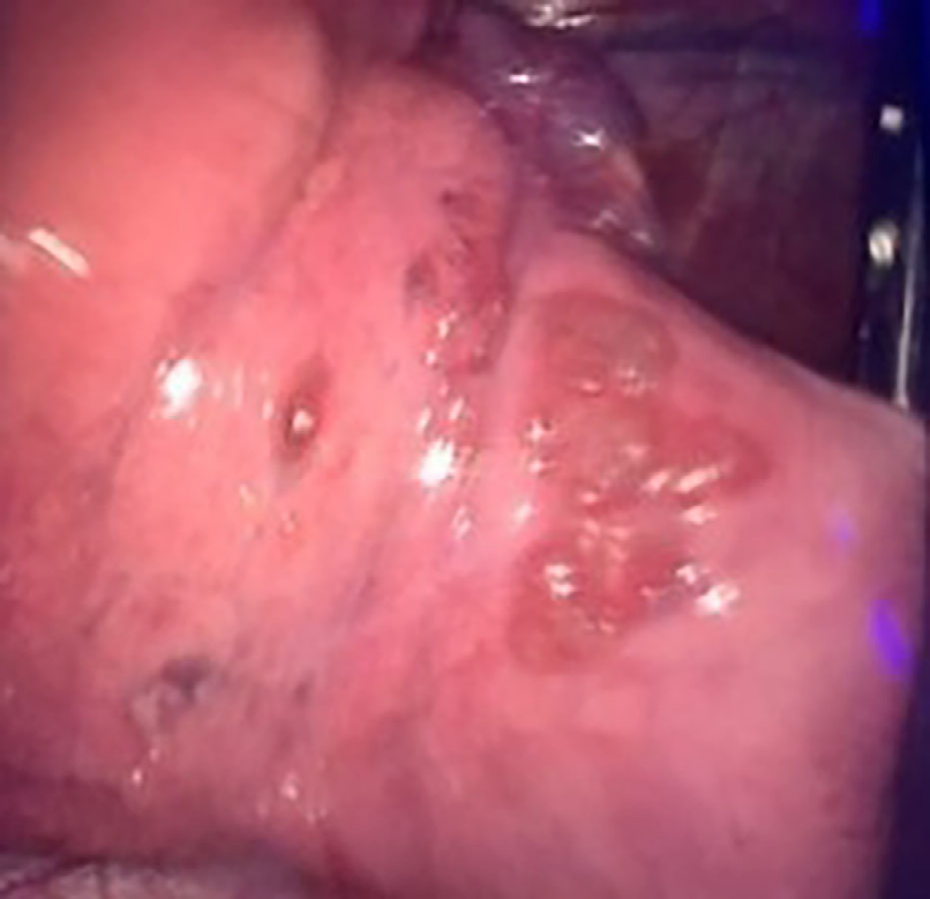
Video‐assisted thoracoscopic surgical (VATS) pleurodesis image demonstrating macroscopic parenchymal cyst formation.

A chest X‐ray revealed a large left‐sided pneumothorax, which required a chest drain to be inserted. This resulted in good symptomatic benefit. A subsequent chest computerized tomography (CT) scan confirmed the pneumothorax, but otherwise demonstrated normal lung parenchyma and no other pleural or chest wall abnormalities. Unfortunately, his pneumothorax failed to resolve following chest drain insertion, and he subsequently required a video‐assisted thoracoscopic surgical (VATS) pleurodesis for definitive management. Macroscopic evidence of cyst formation was evident during the VATS procedure, which suggested a probable secondary spontaneous pneumothorax (SSP) (Fig. 1).

**Figure 2:**
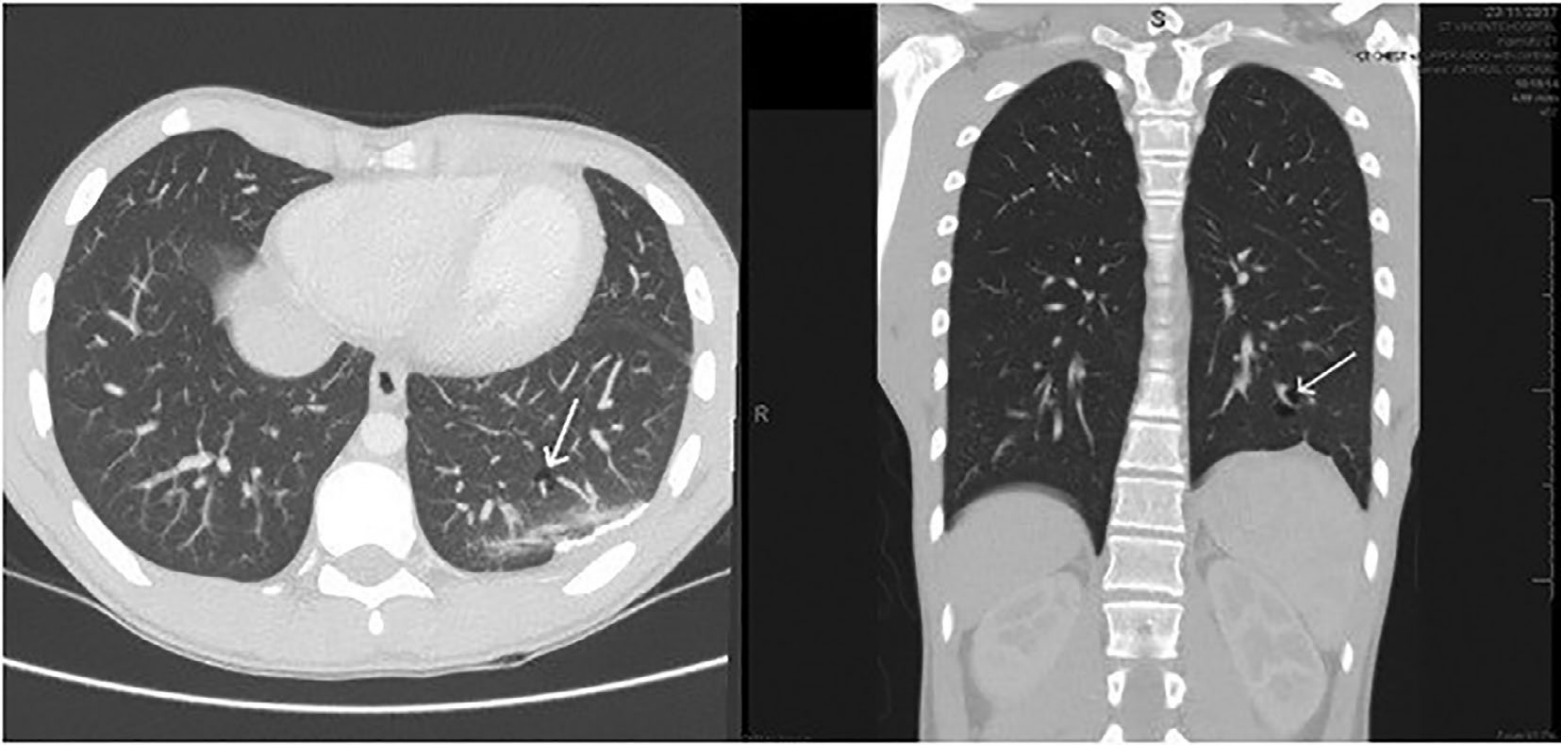
High‐resolution computerized tomography (HRCT) of the chest demonstrating multiple, small, subpleural parenchymal lung cysts.

Following his VATS procedure, a further high‐resolution CT (HRCT) scan of his chest was performed. This scan identified multiple, small, subpleural parenchymal lung cysts, which were not visible on the prior CT scan of his chest (Fig. 2).

A biopsy of the lesion showed pleural bleb and bulla formation, the walls of which were composed of thickened fibrous tissue. Within the involved pleura, patchy chronic inflammatory infiltrate was identified including lymphocytes, histiocytes, and occasional eosinophils. Reactive mesothelial cells were also noted in keeping with subacute/chronic pneumothorax. The histiocytic cells were CD‐68 positive and no infiltrate of CD1a‐positive cells was seen. Following his VATS procedure, he made an excellent recovery and is now back swimming with his high school. Additional genomic sequencing was performed, which demonstrated a germline, splice‐site deletion/insertion mutation (c.249+1_249+2delinsA) in exon 4 of the FLCN gene, confirming a diagnosis of Birt–Hogg–Dubé (BHD) syndrome. Following his diagnosis, a renal ultrasound was performed which demonstrated normal echogenicity, with no focal parenchymal lesion or renal calculus identified.

## Discussion

The incidence of BHD syndrome is unknown, although ~200 families have been identified worldwide. It is an autosomal dominant disorder caused by a germline mutation in the FLCN gene on chromosome 17 (17p11.2), which codes for the protein folliculin. These mutations are small insertion, deletion, splice‐site, and nonsense mutations, which lead in most cases to premature truncation and loss of function of the folliculin protein. The exact role of FLCN is unclear, but it probably functions as a TSG, preventing uncontrolled cell growth and proliferation via the mTOR pathway [1]. This TSG malfunctioning is what likely results in BHD patients having an approximately sevenfold increase in their risk of renal cancers, amounting to a lifetime incidence of ~20% [2].

Clinically, BHD syndrome is characterized by pulmonary cysts, benign skin lesions, and renal tumours [3]. However, these manifestations are highly heterogeneous with some individuals having cutaneous lesions only and some having cutaneous lesions and pulmonary manifestations or cutaneous lesions and kidney tumours. Pulmonary cysts occur in over 80% of individuals, with approximately 30% having a spontaneous pneumothorax in their lifetime, a risk almost 30 times higher than that of the general population. Cysts are usually multiple, bilateral, <1 cm in diameter, and show a subpleural or intraparenchymal distribution. The anticipated findings of an HRCT chest are numerous, irregularly shaped, thin‐walled pulmonary cysts, predominantly <1 cm in size. Additionally, the majority of cysts in patients with BHD syndrome are located in the basilar medial regions of the lungs, which is significantly different from the predominantly apical cysts seen in pneumothoraces secondary to chronic obstructive pulmonary disease (COPD) [3]. Additionally, pulmonary cysts have been demonstrated to contain cytokeratin‐positive, type II pneumocyte‐like cells, which in contrast to the bullae occur secondary to alveolar destruction (e.g. COPD), the BHD pulmonary cysts are slower growing, hamartoma‐like, and at higher risk of rupture. This patient developed a pneumothorax at 15 years, which is highly unusual for BHD syndrome. Pneumothoraces tend to occur in middle age and are rare in patients younger than 20 years [4]. Despite the high frequency of pulmonary involvement, most BHD patients have normal pulmonary function or only a mild obstructive deficit on spirometry [4].

Angiofibromas and fibro‐folliculomas occur in over 90% of patients and appear as round, white‐grey papules in the second or third decade of life, becoming more numerous with age. They can occur anywhere on the head or neck, but predominantly arise from the cheeks or nose. Fibrofolliculomas are a benign hamartoma of the hair follicle and are typically the earliest and most frequent manifestation of BHD syndrome. They often begin to appear in the third decade of life and are derived from epithelial and mesenchymal tissue often easily identifiable on biopsy in the majority of patients. Lesions are usually small, multiple, and diffuse, although lesions as large as 8 mm, with cystic or comedonal structures can occur [5].

Lastly, renal tumours are the most serious complications of BHD syndrome, with a lifetime incidence of ~15–30%. These tumours tend to present at about the age of 50 years (range: 30–70) and are usually slow‐growing malignant tumours. Typically, these are renal chromophobe or oncocytomas, but clear cell carcinoma, papillary carcinoma, and mixed‐type carcinoma can also occur and occasionally can be multiple or bilateral. There are isolated reports of other cutaneous and internal tumours in BHD patients, including lipomas; cutaneous cysts; melanoma, parotid, thyroid, and parathyroid tumours, although these still warrant further validation [6].

Diagnostic criteria for BHD syndrome are based on a combination of clinical features, family history, and confirmation of germline FLCN mutation [1]. Once a diagnosis is confirmed, management is stratified based on the separate sequelae of BHD. There should be a low threshold for chest imaging in any patient with BHD syndrome or a strong family history of recurrent spontaneous pneumothorax, who presents with chest pain or breathlessness. If required, an HRCT scan is preferable over a standard CT chest, as pulmonary cysts in BHD syndrome are often <1 cm in size and as this case report demonstrates, small cysts or those within the lung of occult pneumothoraces can often be missed. However, lung compression from the pneumothorax may have been the cause for the cysts not being seen on the initial standard CT, rather than the scan resolution. Therefore, CT scans for lung cysts should, where possible, only be performed once the lung has fully re‐expanded. Once confirmed, surgical pleurodesis is the preferential approach, due to the high risk of recurrence. Therefore surgical pleurodesis is recommended after a first pneumothorax, unlike with common‐type spontaneous pneumothorax [7]. Skin lesions are benign and often do not cause significant morbidity. Therefore, they often do not require management, but some patients with numerous facial lesions may seek treatment due to cosmetic concerns, often involving carbon dioxide or laser ablation techniques. Lifelong surveillance for renal tumours is required in BHD syndrome, although there is no consensus on the optimal screening timeline. EviQ oncological guidelines suggest baseline abdominal magnetic resonance imaging (MRI) at age 20 years and if no abnormality seen, then three yearly MRI or two yearly high‐resolution ultrasound is continued lifelong [2]. Additionally, renal preservation is of paramount importance for patients with BHD syndrome, given the potential for multiple renal tumours and the risk of developing chronic renal failure following surgery. Therefore, if a renal lesion is confirmed, then active surveillance is adopted unless the lesion is >3 cm, in which case, nephron‐sparing surgery is undertaken [8].

In summary, this case report addresses an unusual, first presentation of a rare familial cancer syndrome in a young patient. Additionally, it highlights key issues surrounding the initial investigations and management of this condition, which differ from standardized guidelines. For example, HRCT is required over conventional CT, which will often miss small lung cysts and that pneumothorax management requires surgical pleurodesis as first‐line management due to the high recurrence risk. Additionally, lifelong surveillance for renal tumours is required in BHD syndrome, although there is no consensus on the optimal screening timeline.

### Disclosure Statement

Appropriate written informed consent was obtained for publication of this case report and accompanying images.
